# CompleteInst: An Efficient Instance Segmentation Network for Missed Detection Scene of Autonomous Driving

**DOI:** 10.3390/s23229102

**Published:** 2023-11-10

**Authors:** Hai Wang, Shilin Zhu, Long Chen, Yicheng Li, Tong Luo

**Affiliations:** 1 School of Automotive and Traffic Engineering, Jiangsu University, Zhenjiang 212013, China; 15830511442@163.com; 2 Automotive Engineering Research Institute, Jiangsu University, Zhenjiang 212013, China; chenlong@ujs.edu.cn (L.C.); liyucheng070@163.com (Y.L.); 3 School of Automobile and Traffic Engineering, Jiangsu University of Technology, Changzhou 213001, China; 15951270769@163.com

**Keywords:** computer vision, autonomous driving, instance segmentation, missed detection

## Abstract

As a fundamental computer vision task, instance segmentation is widely used in the field of autonomous driving because it can perform both instance-level distinction and pixel-level segmentation. We propose CompleteInst based on QueryInst as a solution to the problems of missed detection with a network structure designed from the feature level and the instance level. At the feature level, we propose Global Pyramid Networks (GPN) to collect global information of missed instances. Then, we introduce the semantic branch to complete the semantic features of the missed instances. At the instance level, we implement the query-based optimal transport assignment (OTA-Query) sample allocation strategy which enhances the quality of positive samples of missed instances. Both the semantic branch and OTA-Query are parallel, meaning that there is no interference between stages, and they are compatible with the parallel supervision mechanism of QueryInst. We also compare their performance to that of non-parallel structures, highlighting the superiority of the proposed parallel structure. Experiments were conducted on the Cityscapes and COCO dataset, and the recall of CompleteInst reached 56.7% and 54.2%, a 3.5% and 3.2% improvement over the baseline, outperforming other methods.

## 1. Introduction

In recent years, deep learning has been widely used in various fields, including autonomous driving [1,2], computer vision [3,4,5,6], etc. Object detection [7] and semantic segmentation [8,9,10] are currently the most prevalent algorithms used in the field of autonomous driving. However, these tasks have their own shortcomings: object detection can only identify the location of traffic objects and output a bounding box with location information; it cannot describe its shape in detail. While semantic segmentation is proficient at achieving pixel-level segmentation, it lacks the ability to differentiate between individual instances. The segmentation of objects, including buildings, nature sceneries, and others, exhibits excessive redundancy in the context of autonomous driving.

Therefore, we choose instance segmentation [11] as the juncture between semantic segmentation tasks and object detection tasks. Instance segmentation not only identifies the location of objects of interest but also segments each object of interest pixel by pixel within an image. Consequently, applying the instance segmentation algorithm to the scene of autonomous driving can distinguish pedestrians, cars, riders, and other objects of interest at both the instance and pixel levels, which has engineering significance. The instance segmentation network can output both the bounding box of the object and the segmentation result of the object. The bounding box gives the location information of the objects; the segmentation result can describe the specific shape of the objects. Therefore, we adopt instance segmentation network to study the missed detection problem.

The complexity of autonomous driving scenarios increases the likelihood of the problem of missed detection. Failure to recognize and segment traffic objects, such as cars and pedestrians, during the act of driving can significantly increase the likelihood of life-threatening traffic accidents. Therefore, it is imperative to address the issue of missed detection in order to enhance both the safety and efficiency of traffic.

Existing methods for instance segmentation concentrate on developing new paradigms while ignoring the problems of missed detection. Faster R-CNN [7] is the first two-stage object detection algorithm. First, the bounding boxes are initially screened through the Region Proposal Network (RPN) [7]; then, the network head and NMS post-processing strategies are used to screen them a second time, greatly reducing the number of bounding boxes. Mask R-CNN [12] is an extension of Faster R-CNN, so Mask R-CNN is also the first two-stage instance segmentation network. Mask R-CNN adds a mask head based on Faster R-CNN. During the inference process, the bounding boxes of the detection head are first obtained, then the bounding boxes are aligned to obtain the mask RoI features. Finally, the instance mask is obtained. Since the two-stage instance segmentation algorithm relies heavily on the bounding boxes of the detection head, and the number of the bounding boxes is not guaranteed, it causes the problem of missed detection. In the single-stage instance segmentation network, the SOLO algorithm [13] is relatively typical. SOLO directly predicts instance mask by introducing the concept of “instance categories”, and assigns the “instance categories” to each pixel in the instance according to its location and size. Then, SOLOv2 [14] is proposed, wherein the representation and learning process of the mask are obtained by convolution of dynamically generated convolution kernel parameters and feature maps. The principles of single-stage instance segmentation methods like SOLO and SOLOv2 are the same. The central concept of the single-stage instance segmentation is that a single location corresponds to a single instance mask. This method strikes a good balance between precision and speed, but the concept itself is the source of missed detection. Assuming that the traffic objects are too close, the network determines that centers of traffic objects are in the same location. Consequently, only one instance mask is predicted, and the mask is ambiguous, resulting in missed detection. Multi-stage methods are divided into two types based on whether the attention mechanism [15] is used. The first type is the traditional method that does not use the attention mechanism [15], such as HTC [16] and Cascade R-CNN [17]. This type of algorithm is an extension of the two stages. After each stage, the bounding boxes will be filtered layer by layer and become very few in number, so they are not suitable for solving the problem of missed detection. Owing to the network’s inherent characteristics, the aforementioned traditional instance segmentation methods result in missed detection. This characteristic cannot be altered through structural optimization and, therefore, cannot be used as a baseline to solve the missed detection problem. Therefore, we shift our focus to attention mechanism-based [12] instance segmentation.

Query-based instance segmentation is based on the attention mechanism [12], which treats instances of interest as queries that can be learned. We select QueryInst [18] as our baseline for addressing the missed detection problem. QueryInst is the first query-based instance segmentation method. It proposes using the one-to-one relationship between object query and mask Region of Interest (RoI) features and assigning instance information (shape, location, associative embedding, etc.) contained in the query to the RoI features via dynamic mask heads. During training, QueryInst employs a parallel supervision strategy, wherein gradients computation and parameter updates are simultaneously performed across various stages of the network without mutual interference.

**Figure 1:**
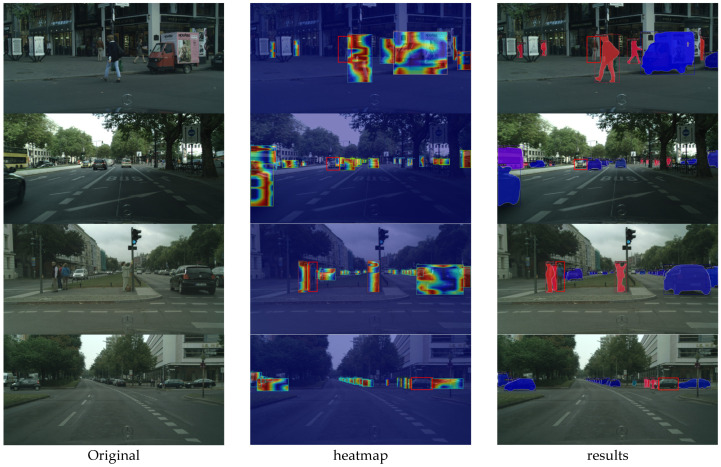
Comparison of QueryInst Class Activation Mapping (CAM) visualization effects for missed detection. The first column is the original image, the second column is the corresponding visual heatmap, and the third column is the results.

We visualized the feature layers with the largest scale of Feature Pyramid Network (FPN) [19] in CAM [20] in order to be able to analyze in detail which factors cause the missed detection. As depicted in Figure 1, we compared the original images, the visualized heat maps, and the final instance segmentation results. The red-circled objects were not detected and segmented due to a detection error in the third column. According to the heat maps, the recognized instance’s position has a maximum heat value. The position of the missed instance, however, has a lower heat value or even no heat value. This indicates that the network has not fully learned the features of the missed instance. The similarities between the missed instance features and background features result in the missed instance features being ignored. Therefore, strengthening the network to extract missed instance features from different levels and completing the missed instance features are essential to resolving the missed detection problem.

Therefore, we propose the CompleteInst algorithm, a multi-stage and efficient perception algorithm based on the attention mechanism that solves the problem of missed detection by strengthening the learning of the features of missed detection instances. Our method solves the problems of missed detection at both the feature and instance levels. At the feature level, we propose global pyramid networks (GPN) and the semantic branch. GPN collects the global information of all instances in each feature layer, strengthens the connection between instances with missed detection and other instances, and completes the global features of missed detection instances. The semantic branch enables the missed instances in the form of RoI to obtain semantic information, enhances the distinction between the missed instances and the background, and completes the missed instances’ semantic features. At the instance level, we propose query-based optimal transport assignment (OTA-Query) by generalizing traditional OTA [21]. OTA-Query augments the number of positive samples for each missed instance, allowing more high-quality positive sample features to participate in the regression of masks and bounding boxes and indirectly completing the missed instance features at the instance level.

Notably, both our semantic branch and OTA-Query are parallel, which is compatible with QueryInst’s parallel supervision mechanism. These methods do not interfere with one another between stages, and they all occur within a single stage. To highlight the parallelism of our structures, we also introduce non-parallel structures as a contrast, utilizing the outcomes of the preceding stage, such as the dynamic interactive module, the enhanced RoI features, etc. Due to their violation of the parallel supervision mechanism, these non-parallel structures did not achieve good results.

The algorithm known as CompleteInst is a query-based instance segmentation method that has been developed by our team. The primary objective of all improvement efforts is to address the issue of missed detection. The evaluation conducted on the Cityscapes and COCO datasets demonstrates that our model surpasses more sophisticated algorithms in terms of performance. The primary contributions of this study are outlined below:

(1) To strengthen the connection between missed detection instances and other instances, we improve FPN and propose GPN. GPN completes the global features of missed detection instances.
(2) To enhance the distinction between the missed instances and the background, the semantic branch is proposed. The semantic branch completes the semantic features of missed detection instances.
(3) To improve the quality of positive samples for each missed instance, OTA-Query is proposed.
(4) To highlight the parallelism of above structures, non-parallel structures are introduced. The parallelism structures are proven to be more effective.

The remainder of the paper is structured as follows: In Section 2, related research on deep learning-based instance segmentation methods is presented. In Section 3, the proposed CompleteInst method’s flow and operating principle are described. The experimental results obtained by the proposed CompleteInst algorithm are analysed in Section 4. Section 5 provides the principal conclusions.

## 2. Related Works

The task of instance segmentation involves the assignment of a mask at the pixel level to each instance of interest within an image, together with the assignment of a class label to each instance. The differentiation between semantic segmentation lies in the requirement to differentiate each individual instance, while the differentiation from object detection is necessary to accurately identify the form of each instance rather than just providing a bounding box output. Hence, instance segmentation entails the simultaneous execution of object detection and segmentation. By employing instance-level discrimination and pixel-level segmentation, the autonomous driving system is able to accurately differentiate between pedestrians, riders, and different categories of vehicles on the road. Instance segmentation methods can be classified into three categories, namely one-stage, two-stage, or multi-stage, depending on the step at which the mask is generated. In light of the recent surge in the popularity of transformers, we also emphasise the significance of query-based methodologies. A comprehensive and elaborate description will be provided for each specific case mentioned subsequently.

### 2.1. One Stage Methods

Dai et al. proposed instance-sensitive FCN [22] to generate proposals using instance-sensitive score maps: firstly, generating a set of instance-sensitive score maps, and then using an assembling module to generate object instances in a sliding window. Wang et al. proposed SOLO [13], which directly predicts instance mask by introducing the concept of “instance categories”, and assigning the “instance categories” to each pixel in the instance according to its location and size. Then, an improved version (SOLOv2 [14]) was proposed, which proposed that the representation and learning process of the mask were obtained by convolution of dynamically generated convolution kernel parameters and feature maps, reducing memory and computation. Xie et al. proposed PolarMask [23], which predicts instance contours through instance center classification and polar coordinate dense distance regression, and utilizes polar centredness to sample high-quality centre samples. Bolya et al. proposed YOLACT [24], which first generates a set of prototype masks, linearly combining coefficients and bounding boxes for each instance, then linearly combines prototypes using the corresponding prediction coefficients, and finally crops using the predicted bounding boxes. Chen et al. proposed BlendMask [25], which includes a blender module, consisting of a bottom module and a top module where the bottom module uses backbone features to predict a set of bases containing low-level semantic information and the top module predicts attention at the instance-level that contains high-level instance information. Du et al. proposed Orient Mask [26], adding an additional OrienHead base on YOLOv3 [27] to predict orientation maps, and combining the obtained bounding boxes and orientation maps for constructing masks. In general, the mask generation method of the single-stage method corresponds to one mask for one position, which is also the reason for the missed detection problem. For example, multiple traffic objects at one location will produce one blurred mask instead of multiple sharp masks.

### 2.2. Two-Stage Methods

The two-stage method usually describes the instance segmentation task as a paradigm of detection first and then segmentation. He et al. proposed Mask R-CNN [12] based on Faster R-CNN [7], adding an additional mask branch and replacing RoIPooling with RoIAlign to improve accuracy. Liu et al. proposed PANet [28], which introduced bottom-up path augmentation, adaptive feature pooling, and fully connected fusion to improve the performance of instance segmentation. Huang et al. proposed Mask Scoring R-CNN [29] to re-score the confidence of the mask by adding a mask IoU branch, which enables the network to predict the IoU of the mask and ground-truth boxes. He et al. proposed the PointRend [30] module to improve Mask R-CNN, using a subdivision adaptive strategy to select N object edge points through MLP processing to iterate and in turn obtain segmentation results. Ke et al. proposed BCNet [31] to model the formation of mask as a combination of two overlapping layers, where the top GCN layer detects occluding objects and the bottom GCN layer infers partially occluded instances to solve the instance occlusion problem. In general, the two-stage method is basically an evolution of Mask R-CNN, which uses the detection branch to generate proposals, then generates mask results from the proposals. The two-stage method is also not suitable for solving the problem of missed detection, because the segmentation performance depends on the performance of the detector. If the detector does not detect the object, the segmentation branch will not output the corresponding mask.

### 2.3. Multi-Stage Methods

Compared with single-stage and two-stage methods, multi-stage methods usually have the highest accuracy. Cai et al. proposed Cascade Mask R-CNN [17], which extended Mask R-CNN to a cascaded structure, and the accuracy was significantly improved. Chen et al. extended Cascade Mask R-CNN and proposed HTC [16] to improve segmentation by constructing mask information flow across each stage and introducing semantic branches. Zhang et al. proposed Refine Mask [32], which fuses fine-grained features in multiple stages to obtain a more refined mask. However, the multi-stage method is essentially an extension of the two-stage method, so it is not suitable for the problem of missed detection, and the inference speed is slow, which is not suitable for industrial deployment.

### 2.4. Query-Based Methods

Query-based methods have become popular in recent years, and the core idea of such methods is to assign instance mask information to learnable queries. SOLQ [33] represents learnable object queries in a unified vector form, while simultaneously performing classification, bounding box regression, and mask encoding. ISTR [34] uses RoI features and image features as the input of the transformer encoder–decoder, and performs N-stage refinement to obtain instance masks. The above methods all have the process of compressing the mask into a vector, which is not conducive to shaping the spatial position information of the missed instances. Mask Transfiner [35] is based on the error-prone tree nodes detected by the transformer, and self-corrects their errors in parallel to refine the quality of the mask. This method focuses on mask refinement and ignores the handling of missed detections. The QueryInst [18] method is to interact the RoI features with the query through the dynamic convolution head to obtain the enhanced RoI features and input them to the box and mask branch in turn to obtain the instance mask. Although the query-based method has no structure that causes missed detection, it does not make any improvement on the missed detection problem. After the above analysis, we chose the QueryInst method as the baseline.

## 3. Methodology

**Figure 2:**
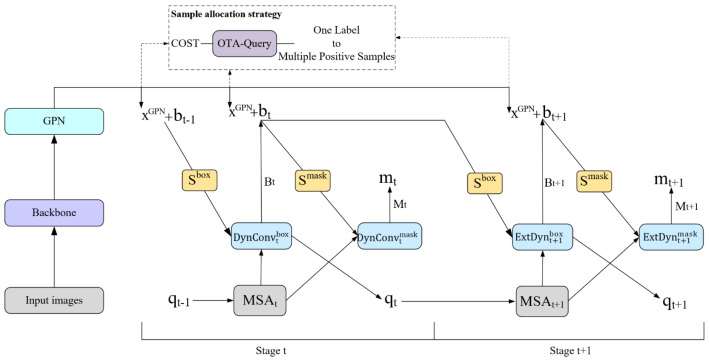
The overall architecture of CompleteInst.

We propose the CompleteInst network based on QueryInst to solve the missed detection problem in autonomous driving scenarios. As Figure 2 shows, the entire network structure consists of three parts: backbone, GPN, and its head. The head is mainly composed of semantic branch (*S^mask^* and *S^box^*), Dynamic Convolution (DynConv), Multihead Self Attention (MSA), and OTA-Query modules. To fully interact with the query, the head is iterated through 6 stages. In the following sections, we introduce the above-mentioned modules in greater depth.

### 3.1. The Overall of CompleteInst

The CompleteInst structure is depicted in Figure 2. First, following backbone and GPN extract and aggregate features, the feature layer *x^GPN^* with four different scales is obtained. Next, *x^GPN^* is fed to the head. The head consists of two parts: the detection head and the segmentation head. The detection head outputs the detection result, and the segment head outputs the segmentation result. The detection head pipeline consists of the following steps:

(1) $$x_{t}^{box}=S^{box}\left(x^{GPN},b_{t-1}\right)$$

(2) $$q_{t-1}^{*}={MSA}_{t}\left(q_{t-1}\right)$$

(3) $$x_{t}^{{box}^{*}},q_{t}={DynConv}_{t}^{box}\left(x_{t}^{box},q_{t-1}^{*}\right)$$

(4) $$b_{t}=B_{t}\left(x_{t}^{{box}^{*}}\right)$$

As described by the above formula, the bounding box $b^{t-1}$ and *x^GPN^* are entered into the semantic branch *S^box^* to get the interested box features $x_{t}^{box}$. The semantic branch enables missed instances to obtain semantic features and complete semantic information. At the same time, the query $q_{t-1}$ input to the t stage is processed by the multi-head self-attention mechanism (${MSA}_{t}$) to obtain the converted query $q_{t-1}^{*}$. ${MSA}_{t}$ correlates query features with each other and removes the isolation between queries. Then, through the dynamic convolution head link $x_{t}^{box}$ and query $q_{t-1}^{*}$, the query $q_{t-1}^{*}$ is achieved by decoding $x_{t}^{box}$. Dynamic convolution decodes RoI features through matrix multiplication, and assigns instance information in query features to RoI features. The enhanced box RoI feature $x_{t}^{{box}^{*}}$ and query $q_{t}\;$which input to the next phase are obtained. $x_{t}^{{box}^{*}}$ is regressed through the detection branch $B_{t}$ to obtain the detection result $b_{t}$ and $b_{t}$ is used for both the input of the next stage and the mask branch for current stage.

The segmentation head pipeline is as follows:

(5) $$x_{t}^{mask}=S^{mask}\left(x^{GPN},b_{t}\right)$$

(6) $$x_{t}^{{mask}^{*}}={DynConv}_{t}^{mask}\left(x_{t}^{mask},q_{t-1}^{*}\right)$$

(7) $$m_{t}=M_{t}\left(x_{t}^{{mask}^{*}}\right)$$

The segmentation pipeline is similar; $b_{t}$ and $x^{GPN}$ are entered into the semantic branch $S^{mask}$ to obtain $x_{t}^{mask}$. The semantic branch enables missed instances to obtain semantic features and complete semantic information. The dynamic convolution head dynamically interacts $q_{t-1}^{*}$ and $x_{t}^{mask}$ to obtain enhanced mask RoI feature $x_{t}^{{mask}^{*}}$ through matrix multiplication. Finally, $x_{t}^{{mask}^{*}}$ is input to the mask branch $M_{t}$, and the *t*-stage instance mask $m_{t}$ is obtained through the full convolution. The query $q_{t}$ and the bounding box $b_{t}$ are fed to the next stage. The above process is iterated six times, refining the bounding boxes and masks.

In the sample assign stage, we adopted the OTA-Query sample allocation strategy, considered the allocation method with the smallest sum of costs to be the Wasserstein distance, applied the Sinkhorn algorithm [36] to solve it, and obtained the results that one label corresponds to multiple positive samples.

Throughout the whole pipeline, GPN, semantic branch, and OTA-Query play an important role in solving the missed detection problem. In the following sections, the implementation and functions of each core component of CompleteInst will be introduced in detail.

### 3.2. Global Pyramid Networks

We propose GPN because the features of missed instances exist in the FPN feature layer. Through up-sampling and the addition of corresponding position elements, the FPN structure performs multi-scale feature fusion. However, this fusion method has certain limitations in that it only considers the correspondence between its own instance features at different scales while ignoring the relationship with other instance features at a greater distance at the same scale. In addition, the acquisition of the feature layers is carried out by superimposing the 3 × 3 convolution in the depth direction, and the scope is also in the form of a local window; therefore, insufficient global information is collected. On the basis of the aforementioned two limitations, we propose GPN and GCN convolution to enhance the acquisition of global features of missed instances and the connection between different instance features at the same scale.

**Figure 3:**
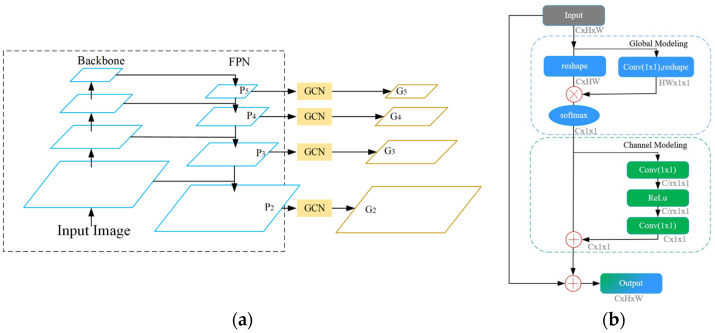
The Global Pyramid Networks and GCN convolution structure. In (**a**), the blue modules within the dotted lines are the backbone network and FPN respectively, and the orange modules are GCN, which outputs feature layers with global information. In (**b**), the input value obtains global information through global modeling (blue modules), and then performs dimension compression through channel modeling (green modules). Finally, it is summed with the original input (the gray) to obtain output of binary information (a fusion of green and blue).

Our GPN are depicted in Figure 3a, where the original FPN is contained within the dashed line. We reconnect the GCN convolution after the original FPN output in order to collect global information for instances that are missed. Finally, global information-containing feature layers G2–G5 are obtained. G2–G5 are considered as inputs for the following six stages. Figure 3b depicts the specific implementation of each step of GCN convolution.

Firstly, the global modelling is performed on feature maps. For the query location *j* in the feature maps, the attention weight of location *j* is first obtained through 1 × 1 convolution. It is multiplied by the feature corresponding to the *j* location, and then sums all the query positions (matrix multiplication) to obtain the corresponding global features $\sum_{j}a^{j}x^{j}$. Then, SoftMax normalization is performed. In order to reduce the calculation consumption caused by the increase of the number of channels in the deeper layer and get channel information, we set the 1 × 1 convolution in channel modelling, so that the number of channels after the convolution is C/r, where r is the bottleneck ratio. The selection and comparison of the r value are conducted in the subsequent experimental phase. The obtained global features have channel dependencies. Finally, the broadcast mechanism is used to add element locations to complete global features.

### 3.3. Semantic Branch

The semantic branch is proposed due to the presence of missed instances’ features within the RoI features. The semantic branch is incorporated to generate semantic features, providing semantic information for missed instances and enhancing the differentiation between these instances and the backdrop. In the semantic branch, we also include another head to supervise the semantic features so that our semantic information is explicit. The final step is to combine the RoI with semantic features with the original RoI with global features to obtain more comprehensive instance features.

The formula expresses the specific process:

(8) $$x^{sema}=PPM\left(conv\left(G_{2}\right)\right)$$

(9) $$x_{box}^{t}=P^{box}\left(x^{GPN},b^{t-1}\right)+P^{box}\left(x^{sema},b^{t-1}\right)$$

(10) $$x_{mask}^{t}=P^{mask}\left(x^{GPN},b^{t}\right)+P^{mask}\left(x^{sema},b^{t-1}\right)$$

**Figure 4:**
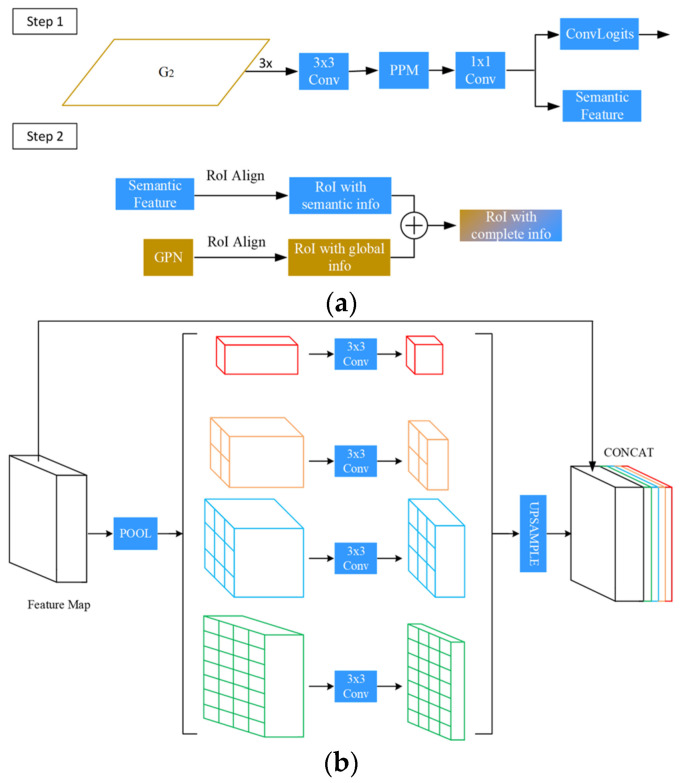
The architecture of the semantic branch and PPM. In (**a**), the semantic features (blue module) are obtained through the integration of PPM and convolution; Then, through the RoI alignment operation, the semantic features with semantic information and the semantic features with global information are The GPN features (orange and yellow modules) are added together, and finally the RoI features (yellow and blue) with complete information are obtained. In (**b**), after the features are pooled, four different scale features are obtained (the red has the largest scale, followed by the orange, then the blue, and finally the green is the smallest), and then fused through upsampling and splicing operations to obtain different Characteristics of scale.

Figure 4a illustrates the particular structure of our semantic branch. We use the G2 layer as input in the first step because this layer contains more specific information and integrates high-level semantic information, resulting in richer information. After three 3 × 3 convolutions, the Pyramid Pooling Module (PPM) module [37] is introduced in order to obtain semantic features with varying scales. Figure 4b portrays the PPM module. Specifically, the feature layer is divided into 6 × 6, 3 × 3, 2 × 2, and 1 × 1 grids, then each grid is averaged and pooled, and the pooled results are aggregated by up-sampling. A 1 × 1 convolution is then used to adjust the channel dimension to match the dimension of the original RoI features. Semantic predictions are derived by the process of logical convolution. To ensure the quality of the semantic features generated by the succeeding branches, we apply cross-entropy loss on the predictions and compare them with the semantic segmentation labels.

The second step involves performing feature fusion. We use bounding boxes to align semantic features to obtain RoI semantic features. In the same way, we align the original GPN to obtain the RoI global features and add the elements of the two to obtain the more comprehensive RoI features for subsequent dynamic interaction and regression.

### 3.4. OTA-Query

**Figure 5:**
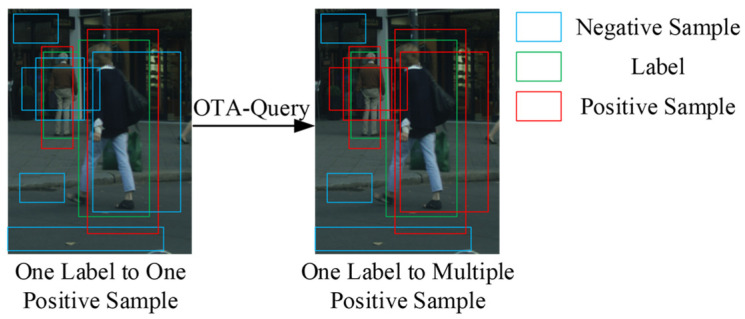
Comparison between bipartite matching and OTA-Query sample allocation.

QueryInst’s sample allocation strategy corresponds one label to one positive sample. This one-to-one optimal allocation problem is treated as a bipartite matching by QueryInst, which employs the Hungarian algorithm to solve it. However, the current allocation strategy of assigning one label per positive sample is suboptimal for addressing the missed detection problem. It would be more suitable to adopt an allocation strategy of assigning one label per multiple positive samples. As depicted in Figure 5, under the one-to-one sample allocation, the old man in the back is incorrectly identified (as depicted in Figure 1), indicating that a single positive sample for regression is insufficient to improve the quality of features for missed instances. Therefore, we present OTA-Query to match missed instances with multiple positive samples and enhance the network’s extraction of missed instance features based on the number of positive samples.

The OTA-Query sample allocation strategy is designed. Specifically, the label is considered the supplier, and a specific number of $s_{i}=k$ labels are assigned. Considering the samples as the demander, a positive sample seeks a label. In addition, the background class is considered a unique supplier, as it supplies the “background label.” Suppose there are m labels, and each label can provide *k* labels of its own number to assign to positive samples in an image. This image contains n boxes, each of which will receive a label. Positive samples are those that successfully match the label, while the remaining *n* − *k* × *m* samples are assigned to the background class and become negative samples. Consequently, the following target formula is presented:

(11) $$min\sum_{i=1}^{m}\sum_{j=1}^{n}c_{ij}\pi_{ij}$$

(12) $$s.t.\sum_{i=1}^{m}\pi_{ij}=1,\sum_{j=1}^{n}\pi_{ij}=s_{i},\sum_{i=1}^{m}s_{i}=n,$$

(13) $$\pi_{ij}\geq 0,i=1,2\ldots ,m,j=1,2\ldots ,n$$

The optimal transfer problem involves the determination of the Wasserstein distance, which is defined as the minimal total cost incurred in transferring each label to its corresponding sample. *i* represents the label index and j represents the sample index. $\pi_{ij}$ indicates the *i*-th label supplied to the *j*-th sample. $c_{ij}$ represents the cost of supplying the *i*-th label to the *j*-th sample. The specific cost calculation method is as follows:

(14) $$c_{ij}={\alpha L}_{cls}\left(i,j\right)+\beta *L_{reg}\left(i,j\right)+\gamma *L_{GIOU}\left(i,j\right)\left(positive\;sample\right)$$

(15) $$c_{ij}={\alpha L}_{cls}\left(i,j\right)\left(negative\;sample\right)$$

The cost is the weighted sum of classification loss, regression loss, and GIoU loss, assuming the sample is a positive sample. The weight coefficients *α*, *β*, and *γ* adhere to the setting of the loss function, which are 2, 5, and 2, respectively. If the sample is negative, the cost is restricted to classification loss. To determine the *k* value, we calculate the IoU between the sample and the label, select the top 10 IoU values for each label, add them, and round them up. To obtain the optimal matching result for the solution of the target formula in Equation (6), the Sinkhorn algorithm for iterative calculation is used.

### 3.5. Parallel and Non-Parallel

**Figure 6:**
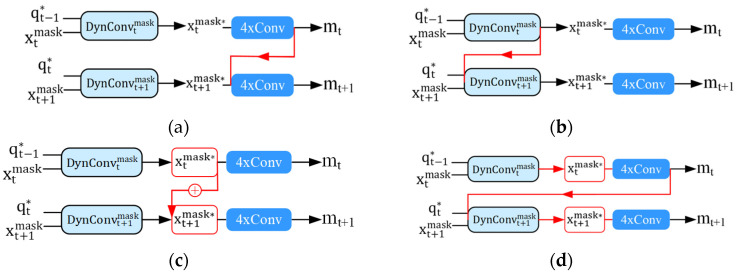
Four non-parallel mask serial interaction structures. (**a**) Serial interactive of mask head convolution sequence. (**b**) Serial interaction of dynamic mask interactive module. (**c**) Serial stacking of enhanced mask features. (**d**) Overall serial interactive of the whole mask head.

Our semantic branches and OTA-Query are parallel. All of their implementations occur within the stage, which is compatible with the parallel supervision mechanism of QueryInst, thereby enhancing the extraction of missed instance features. In each stage, the RoI features with semantic information extracted by the semantic branch will be fused with the original RoI features. In addition, OTA-Query will perform one-to-multi sample allocation at each stage. The preceding stage and the final stage of the structure described above do not interfere. To demonstrate the superiority and efficacy of the aforementioned parallel structure, we propose a non-parallel structure in which different stages influence and interact with one another for comparison. As depicted in Figure 6, we made four modifications to the QueryInst algorithm for the serial interaction of the mask branch.

The overall logical framework is depicted in Figure 6. These four structures are the serial interaction across stages. The first structure indicates that the four convolution sequences at the same location in the previous stage are fully utilized before the convolution of the current stage. This is described with the formula:

(16) $$m_{t}=M_{t}\left[M_{t-1}\left(x_{t}^{{mask}^{*}}\right)\right]$$

where the $x_{t}^{{mask}^{*}}$ represents the enhanced mask feature. The $x_{t}^{{mask}^{*}}$ is continuously input to the mask heads of the *t* − 1 and *t* stages, and finally the segmentation result $m_{t}$ is obtained.

The second structure makes extensive use of the dynamic mask interactive module of the previous stage. This is described with the formula:

(17) $$x_{t}^{{mask}^{*}}={DynConv}_{t}^{mask}\left[{DynConv}_{t-1}^{mask}\left(x_{t}^{mask},q_{t-1}^{*}\right)\right]$$

where $x_{t}^{mask}$ represents the mask feature and $q_{t-1}^{*}$ represents the enhanced query feature. $x_{t}^{mask}$ and $q_{t-1}^{*}$ are input to the dynamic heads of the *t −* 1 and *t* stages continuously, and finally the $x_{t}^{{mask}^{*}}$ is obtained.

The third structure is the fusion of enhanced mask features between stages. This is described with the formula:

(18) $$\begin{array}{c} x_{t}^{{mask}^{*}}=x_{t-1}^{{mask}^{*}}+x_{t}^{{mask}^{*}} \end{array}$$

where $x_{t}^{{mask}^{*}}$ and $x_{t-1}^{{mask}^{*}}$ represent the enhanced mask feature of the *t −* 1 and *t* stages, respectively. The sum of $x_{t-1}^{{mask}^{*}}$ and $x_{t}^{{mask}^{*}}$ results in the new $x_{t}^{{mask}^{*}}$.

The fourth structure is to unify the first three non-parallel structures, and the complete the serial interaction of the mask branches. The formula is expressed as:

$$m_{t}^{\prime }=M_{t-1}[{DynConv}_{t-1}^{mask}\left(x_{t}^{mask},q_{t-1}^{*}\right)+x_{t-1}^{{mask}^{*}}],$$

(19) $$m_{t}=M_{t}[{DynConv}_{t}^{mask}\left(m_{t}^{\prime },q_{t-1}^{*}\right)]$$

This can be expressed as the $x_{t}^{mask}$ feature is first input to the *t* − 1 stage to obtain the $m_{t}^{\prime }$ result, then the $m_{t}^{\prime }$ result is input to the t stage to obtain the final $m_{t}$.

### 3.6. Loss Function

To supervise the semantic branch based on the baseline, we added an additional loss function. Consequently, the overall loss function comprises the following components:

(20) $$L=\sum_{t=1}^{T}\lambda_{cls}L_{t}^{cls}+\lambda_{reg}L_{t}^{reg}+{\lambda_{giou}L}_{t}^{giou}+\beta L_{t}^{mask}+\gamma L^{sema}$$

For the detection branch, we adhered to the hyperparameter settings of Sparse R-CNN [38], where $\lambda_{cls}$, $\lambda_{reg}$, and $\lambda_{giou}$ are 2, 5, and 2, respectively. We adopted Focal Loss [18] as the category loss function $L_{t}^{cls}$, and L1 Loss and GIoU Loss are utilized as the bounding box loss functions $L_{t}^{reg}$ and $L_{t}^{giou}$, respectively. For the segmentation branch, we followed the hyperparameter settings of QueryInst [18], where *β* is 8, and $L_{t}^{mask}$ adopts Dice Loss. For supervision of the semantic branch, we used the cross-entropy loss function, with the following formula, where s is the semantic segmentation result and $\hat{s}$ is the label. The ablation experiment section discusses the selection of the *γ* parameter.

(21) $$L_{sema}=CE\left(s,\hat{s}\right)$$

## 4. Experiments and Results

### 4.1. Experiment Details

We used the COCO [39] and Cityscapes [40] instance segmentation datasets for experiments. The evaluation metric utilized the standard mask AP, whereas the detection section utilized the box AP.

Cityscapes: We performed hyperparameter tuning, ablation experiments, and comparisons with other algorithms on the Cityscapes dataset. Cityscapes is a city-centric traffic scene dataset, and its instance segmentation part contains 8 categories, 2975 training images, 500 validation images, and 1525 testing images. The images are of higher resolution and have finer labels than COCO. In order to ensure that the labels used by the parallel semantic branch are of the semantic segmentation type, we additionally utilized labels corresponding to semantic segmentation for 2975 images. By default, the semantic segmentation label has 34 categories, but in previous research, there was a mapping from 34 categories to 19 categories, so there were two kinds of labels. Thus, we also introduced the number of categories into the experimental comparison part.

COCO: To demonstrate the generalization of our method, we also used CompleteInst on the COCO dataset for experiments. We used the COCO train2017 split (118,000 images) for training and the val2017 split (5000 images) as validation. In addition, for the parallel semantic branch, we satisfied the training requirement by adding an additional 2017 COCO-Stuff dataset [41]. The labels of 2017 COCO-Stuff dataset contain a total of 172 categories: 80 things, 91 stuff and 1 ‘unlabeled’. The ‘unlabled’ class corresponds to instances where the pixel value is 255.

Training: We put the model on two 3090s with 24G video memory for training. Due to limited computational resources, we only made a case in which the backbone is ResNet-50-FPN [42]. For the Cityscapes dataset, the number of training epochs was set to 64, the number of batches was 8, and the initial learning rate was set to 1.25 × 10^−5^, which was reduced to 1/10 at the 56th epoch. Since the objects in the autonomous driving scene are relatively dense, we adopted 300 proposal boxes and object queries to pursue better performance. For the COCO dataset, the number of training epochs was 12, the initial learning rate was set to 0.0001, and the number of batches was 16, which were reduced to 1/10 in the 11th and 12th rounds, respectively. The number of proposal boxes and object queries was set to 100. We used both 6-stage and AdamW [43] optimizers with the weight decay of 0.0001.

Inference: Given an input image, after GPN, semantic branching, dynamic interaction, and head regression, output the top 100 bounding box predictions and corresponding instance masks in the final stage, without Non-Maximum Suppression. Inference speed was tested using a single 3090. For a single image of the COCO dataset, the length and the width are 1333 and 800 respectively. For the Cityscapes dataset, a single image is 2048 length and 1024 width.

Evaluation Metrics: In order to more accurately evaluate the missed detection performance of the model, we gave the average recall (*AR*) as a metric. Recall refers to the proportion of positive examples successfully recognized by the model among all positive examples. It focuses on the model’s recall for each category, then averages these recalls, and finally gets the average recall. The calculation formula for *AR* is as follows:

$$R=\frac{TP}{TP+FP}$$

where *R* represents the recall rate of a single category, *TP* represents the number of correctly predicted positive examples, and *FP* represents the number of incorrectly predicted positive examples. The denominator is the sum of *TP* and *FP*, expressed as the number of all positive examples. *TP* is divided by *TP* + *FP* to get the proportion of positive examples correctly predicted by the model.

(22) $$AR=\sum_{1}^{i}R$$

The category average of the recall of all categories is taken to get the average recall, as shown in the formula, where *AR* represents the average recall and *i* represents the number of categories.

### 4.2. Experimental Results

#### 4.2.1. Evaluation Results on the Cityscapes Dataset

We compared CompleteInst with prevalent instance segmentation algorithms in recent years, such as QueryInst [18], Mask R-CNN [12], SOLOv2 [14], CondInst [44], PointRend [30], HTC [16], SOLQ [33], and ISTR [34]. The results of each method on the Cityscapes validation set are presented in Table 1. Our CompleteInst increased the mask AP by 1.9 points when compared to the baseline and 1.5 points when compared to the multi-stage cascade method HTC. The mask AP is 2.5 and 2.6 points greater than the query-based methods SOLQ and ISTR. In comparison to the two-stage techniques Mask R-CNN and PointRend, the mask AP is 4.4, and 3 points higher, respectively. The mask AP is 4.7 and 3.6 points greater than the single-stage methods SOLOv2 and CondInst, respectively. The average recall (AR) of CompleteInst reaches 56.7, which is 3.5 points higher than the baseline, and the average recall (AR) of CompleteInst is typically greater than other methods. This shows that the recall rate of CompleteInst’s detection and segmentation is much higher than other methods. CompleteInst is effective in solving the problem of missed detection. Our method achieves good results for each category’s mask AP, with the exception of trucks and buses, where it performs marginally worse than the HTC and SOLOv2 methods.

**Figure 7:**
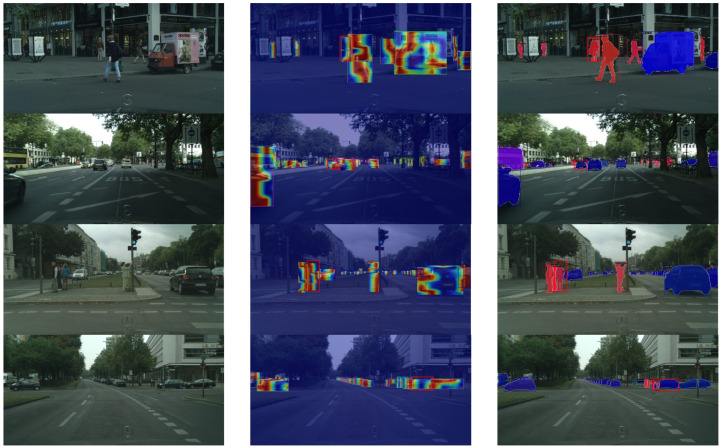
Comparison of CompleteInst CAM visualization effects for missed detection. The first column is the original image, the second column is the corresponding visual heatmap, and the third column is the result. The features of the missed instances have been completed, and the heat map values have reached normal levels.

Figure 7 depicts the outcomes of our methodology. All of these images are from Figure 1. Figure 1 demonstrates that the baseline fails to recognize the instance at the red-circled location, whereas our method can detect and segment it with precision. In accordance with the heat map, the missing instances’ heat value has returned to normal, which is significantly different from the background features. This demonstrates that our method completes the features of missing instances and improves the network’s ability to extract missing instance features.

**Figure 8:**
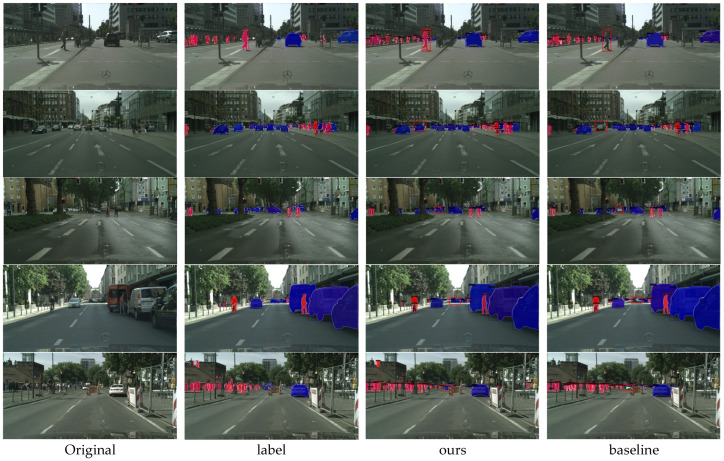
Comparison of CompleteInst and baseline effects. The CompleteInst algorithm has been greatly improved compared to the baseline, and almost all missed instances have been detected. The effect achieved by the algorithm is close to the label level.

Comparing the segmentation performance of our method to baselines on the validation and training sets is depicted in Figure 8. At the position denoted by the red circle, it is evident that some objects in the baseline segmentation results, including the pedestrian in the middle of the first row and the car on the left side of the second row, have not yet been segmented. Our method completes the missing instance’s features so that it contains more foreground information. Essentially, the image’s object of interest is entirely segmented. Therefore, our method solves the problem of missed detection.

Based on the domain difference-based evaluation method proposed in [45], we compared CompleteInst with the baseline to evaluate the effect on the test set. The results are shown in Table 2. Among the three sets of experimental data, CompleteInst has a relatively smaller Fréchet Distance compared to the baseline, which shows that CompleteInst is more suitable for detecting unknown autonomous driving environments and is less prone to missed detection problem. From the test set, the prediction accuracy of the CompleteInst method is much higher than the baseline prediction accuracy value. This is consistent with the Fréchet Distance value. Finally, we give the variance value between Ground_truth Accuracy and Prediction Accuracy. Obviously, CompleteInst reaches 3.69, which is lower than 4.05. This shows that the results predicted by our model are more stable, and therefore more suitable to solve the missed detection problem of unknown scenarios.

#### 4.2.2. Evaluation Results on the COCO Dataset

In order to demonstrate the generalizability of our model, we conducted validation set experiments on the COCO dataset and compared our results to those of other methods. Table 3 displays the specific results. Our CompleteInst mask achieves 38.9 mask AP, which is 1.5 AP better than the baseline, and 43.5 box AP, which is 1.9 AP better than the baseline, based on 12 training epochs. The speed is 1.2 frames per second (FPS) slower than the baseline, but we believe this is acceptable given the significant improvement in baseline precision. In comparison to HTC, SOLQ, and ISTR methods, the accuracy is superior, and the mask accuracy is increased by 1.5 AP, 0.2 AP, and 0.3 AP, respectively. In addition, our CompleteInst achieves excellent performance with an average recall (AR) of 54.2 AR, which is significantly superior to all other methods. This shows that CompleteInst is effective in solving the missed detection problem on the COCO dataset. Our method achieves 41.7 AP and 58.7 AP for medium and large objects, an improvement of 1.4 AP and 2.5 AP over the baseline and a significant lead over all other methods. Our method improved by 1.0 AP for small targets compared to the baseline, but it is not optimal. The ISTR is more accurate for small objects, which is an area that requires further development. In terms of detection precision, our method is weaker than SOLQ and ISTR, an area where we must also improve.

### 4.3. Ablation Experiment

#### 4.3.1. Study of the Global Pyramid Networks

In order to demonstrate the efficacy of GPN, we compared the GPN structure to the baseline, and the results are presented in Table 4. The use of GPN results in greater accuracy than the baseline. AP reaches 39.4 and AR reaches 54.3 when the bottleneck ratio r value of GCN is set to 4. The AP and AR accuracies are improved by 0.5 and 1.1, respectively, compared to the baseline. This demonstrates that the GPN structure is effective for addressing the problem of missed detection. GPN strengthens the connection with other instances by establishing the global context relationship between missing instances and other positions on the feature map, then completes the missing instances’ global features. It is beneficial for the network to extract the global features of missing instances.

#### 4.3.2. Study of the Semantic Branch

To demonstrate the efficacy of the semantic branch, we compared the semantic branch structure to the baseline, and the results are shown in Table 5 and Table 6. The addition of the semantic branch improves the accuracy of the baseline, as observed. In addition, we compared the number of semantic branch categories and the value of the weight coefficient *γ*. The addition of a semantic branch improves AP and AR by 0.8 and 1.5 points, respectively, when the number of categories and *γ* are 19 and 0.3, respectively, compared to the baseline. This indicates that the semantic branch makes the semantic information of instances that have been missed more prominent and completes their semantic features.

#### 4.3.3. Study of the OTA-Query

To demonstrate the efficacy of OTA-Query, OTA-Query was compared to the baseline bipartite match method, and the results are shown in Table 7. OTA-Query is observed to improve the accuracy of the baseline. AP and AR improved by 1.1 and 1.8, respectively, compared to the baseline. This demonstrates that, for the missed detection problem, a single label corresponding to multiple positive samples is appropriate. OTA-Query enhances the quality of positive samples of missed instances by increasing their quantity. It indirectly improves the network’s extraction of missed instance features at the instance level.

#### 4.3.4. Study of the CompleteInst

To demonstrate the efficacy of our proposed method and the independence of the accuracy gains contributed by each component, we conducted ablation experiments on each component. The particular ablation studies are presented in Table 8. The results indicate that the introduction of GPN increases AP and AR by 0.5 and 1.1, respectively, when compared to the baseline. It is demonstrated that GPN enhances the global information of missed instances. Based on this, we introduced the semantic branch, which further improved AP and AR by 1.1 and 2.1, respectively. This demonstrates that the semantic branch enhances the semantic information of missed instances at the feature level of the RoI. Finally, we replaced the bipartite matching with the OTA-Query sampling method, bringing the AP and AR to 40.8 and 56.7, an increase of 1.9 and 3.5 points, respectively. By improving the quality of positive samples of missed instances, OTA-Query indirectly improves the network’s ability to extract the features of missed instances. Overall, our method solves the problem of missed detection from three distinct perspectives. Our method is, therefore, effective for handling missed detection scenarios in autonomous driving.

#### 4.3.5. Study of the Parallelism and Non-Parallelism

To demonstrate that our proposed branches possess the parallelism property, we experimentally validated the four non-parallel methods described in Section 3.5. The experimental results of the non-parallel structures are presented in Table 9. The structures are generally ineffective at enhancing QueryInst, and the AP is decreased relative to the baseline. Specifically, the 6-c structure in Section 3.5 has the least detrimental effect, the AP is unaffected, and the AR decreases by 0.8. AP and AR decreased by 1.1 and 1.9, respectively, as a result of the 6-d complete mask branch serial interaction, which had the greatest impact. Structures 6-a and 6-b also degraded the baseline’s AP and AR. The experimental results demonstrate that the interaction structure involving multiple stages destroy the parallel supervision mechanism of QueryInst, disrupting the reverse transfer of gradient information, and diminishing precision. Therefore, the non-parallel approach is not recommended. The semantic branch and OTA-Query adhere to the mechanism of parallel supervision without interfering with the gradient flow of different stages; the occurrence stages are all contained within it, and the stages do not influence one another. Our CompleteInst is therefore effective in parallel.

## 5. Conclusions

In this paper, we improved the query-based instance segmentation method QueryInst and proposed a novel CompleteInst network for the missed detection problem in autonomous driving. From various perspectives, we proposed a more comprehensive solution strategy. We proposed GPN to collect the global information of missed instances and to strengthen the connection between missed instances and other location instances; we then proposed the semantic branch, which combines RoI global features obtained from GPN alignment with RoI semantic features to obtain more comprehensive RoI features. Finally, we presented the OTA-Query sample allocation strategy at the instance level, which enhances the quality of positive samples of missed instances and indirectly improves the extraction of features from missed instances. The parallel nature of our semantic branch and OTA-Query is compatible with the parallel supervision mechanism of the baseline. In order to further demonstrate the superior performance of the proposed parallel structure, we also compared it with non-parallel structures.

Overall, our CompleteInst yields favourable outcomes and is vastly superior to the baseline. It provides a unique solution to the occlusion problem of autonomous driving. However, CompleteInst has the disadvantage that the real-time performance of the algorithm is slightly poor and the accuracy of a single category is not optimal. This is a direction worthy of further improvement. We hope that this work will complement the extension and application of instance segmentation to the missed detection scenario of autonomous driving.

## Figures and Tables

**Table 1:** Comparison of main results on Cityscapes dataset. Among them, AP and AR represent the algorithm precision and recall of all categories respectively. The next eight columns are precision comparisons for a single category. Except for trucks and buses, all other metrics outperform other algorithms.

Method	AP	AR	Person	Rider	Car	Trunk	Bus	Train	Mcycle	Bicycle
Mask R-CNN	36.4	53.1	34.8	27.0	49.1	30.1	40.9	30.9	24.1	18.7
SOLOv2	36.1	52.4	30.8	27.0	45.8	38.7	62.3	42.2	19.8	22.4
CondInst	37.2	50.5	36.3	28.5	55.7	37.6	57.8	38.2	20.6	23.3
PointRend	37.8	51.4	36.8	28.5	56.9	37.0	58.9	40.5	20.0	23.8
HTC	39.3	56.0	38.0	30.9	57.1	40.5	59.7	40.5	22.5	25.0
SOLQ	38.3	55.8	37.6	29.8	56.8	35.5	57.7	43.7	21.4	24.2
ISTR	38.2	54.1	37.0	30.0	57.3	35.6	58.1	42.5	20.8	23.9
Baseline	38.9	53.2	38.5	30.5	57.1	35.3	58.0	44.7	21.9	24.8
Ours	40.8	56.7	40.1	32.3	59.9	36.8	61.1	45.9	24.8	26.7

**Table 2:** Comparison results of unlabelled test sets based on domain differences. Ground_truth Accuracy is expressed as the accuracy of the validation subset, Fréchet Distance is expressed as the domain difference value, and Prediction Accuracy is expressed as the accuracy of each test subset. RMSE [45] is expressed as the size of the variance between accuracies.

Group	Ground_Truth Accuracy		Fréchet Distance		Prediction Accuracy		RMSE	
	Baseline	Ours	Baseline	Ours	Baseline	Ours	Baseline	Ours
1	38.6	40.7	19.1	15.4	37.3	42.1	4.05	3.69
2	38.3	40.1	19.6	15.6	39.5	41.9		
3	37.5	39.2	20.4	16.0	36.2	41.2		

**Table 3:** Comparison of COCO verification set results. AP^bbox^ is expressed as the detection precision of bounding boxes. AR is expressed as the detection recall of bounding boxes. APS, APM, and APL represent the segmentation precision of small targets, medium targets, and large targets, respectively. FPS is expressed as the inference speed of the algorithm. Except for AP^bbox^ and inference speed, all other metrics were the best among the tested methods.

Method	Epoch	AP^bbox^	AP	AR	APS	APM	APL	FPS
Mask R-CNN	12.0	38.2	34.7	50.7	18.3	37.4	47.2	24.1
SOLOv2	12.0	-	34.8	49.9	13.4	37.8	53.7	25.4
CondInst	12.0	39.7	35.7	48.3	17.1	39.1	50.2	28.6
PointRend	12.0	38.4	36.3	49.1	19.8	39.4	48.5	16.2
HTC	12.0	42.3	37.4	51.4	19.6	40.4	51.7	6.2
SOLQ	36.0	46.2	38.7	50.0	20.2	41.1	51.5	15.0
ISTR	36.0	46.8	38.6	50.9	22.1	40.4	50.6	13.8
Baseline	12.0	41.6	37.4	51.0	17.9	40.3	56.2	16.0
Ours	12.0	43.5	38.9	54.2	18.9	41.7	58.7	14.8

**Table 4:** Effect of Global Pyramid Networks. ∆AP and ∆AR are expressed as the difference from baseline precison and recall. GPN works best when the ratio is 4.0.

Type	Ratio	AP	∆AP	AR	∆AR
FPN	w/o	38.9		53.2	
GPN	16.0	39.2	+0.3	54.0	+0.8
GPN	4.0	39.4	+0.5	54.3	+1.1

**Table 5:** The effect of the number of semantic branch categories, w/o expressed as baseline. The semantic branch achieves the best effect when the number of categories is 19.0.

Num of Categories	AP	∆AP	AR	∆AR
w/o	38.9		53.2	
19.0	39.7	+0.8	54.7	+1.5
34.0	39.4	+0.5	53.7	+0.5

**Table 6:** The effect of semantic branch γ coefficient. The accuracy is maximized and the effect is best when the *γ* coefficient is 0.3.

γ	AP	∆AP	AR	∆AR
w/o	38.9		53.2	
0.2	39.0	+0.1	53.8	+0.6
0.3	39.7	+0.8	54.7	+1.5
0.4	39.3	+0.4	54.1	+0.9

**Table 7:** Effect of OTA-Query. The OTA-Query sampling strategy is better than the baseline binary matching.

Sampling Strategy	AP	∆AP	AR	∆AR
Bipartite match	38.9		53.2	
OTA-Query	40.0	+1.1	55.0	+1.8

**Table 8:** Ablation studies on the proposed CompleteInst. GPN, semantic branch, and OTA-Query all further improve the algorithm performance, reaching 40.8 AP and 56.7 AR, which means that the effects of these modules are cumulative. The symbol “√” indicates modules that have been used or owned.

Baseline	GPN	Semantic Branch	OTA-Query	AP	∆AP	AR	∆AR
√				38.9		53.2	
√	√			39.4	+0.5	54.3	+1.1
√	√	√		40.0	+1.1	55.3	+2.1
√	√	√	√	40.8	+1.9	56.7	+3.5

**Table 9:** Comparison of non-parallel completion and parallel completion. All non-parallel structures worsen algorithm performance. This shows that non-parallel structures are undesirable.

Complete Classification	Method	AP	∆AP	AR	∆
Baseline	/	38.9		53.2	
Non-parallel	6-a	38.7	−0.2	50.7	−2.5
	6-b	38.3	−0.6	51.8	−1.4
	6-c	38.9	0	52.4	−0.8
	6-d	37.8	−1.1	51.3	−1.9

## Data Availability

Data are contained within the article.
